# CCL20 Promotes Ovarian Cancer Chemotherapy Resistance by Regulating ABCB1 Expression

**DOI:** 10.1247/csf.18029

**Published:** 2019-02-14

**Authors:** Shan Su, Xueqin Sun, Qinghua Zhang, Zhe Zhang, Ju Chen

**Affiliations:** 1 Department of Gynecology, the Central Hospital of Zibo, Zibo 255000, Shandong, China; 2 Department of Ultrasound, the Central Hospital of Zibo, Zibo 255000, Shandong, China

**Keywords:** CCL20, ovarian cancer, doxorubicin resistance, tumor-promoting, ABCB1

## Abstract

Ovarian cancer (OC) is one of prevalent tumors and this study aimed to explore CCL20’s effects on doxorubicin resistance of OC and related mechanisms. Doxorubicin-resistant SKOV3 DR cells were established from SKOV3 cells via 6-month continuous exposure to gradient concentrations of doxorubicin. Quantitative PCR and Western blot assay showed that SKOV3 DR cells had higher level of CCL20 than SKOV3 cells, and doxorubicin upregulated CCL20 expression in SKOV3 cells. MTT and cell count assay found that CCL20 overexpression plasmid enhanced doxorubicin resistance of SKOV3 and OVCA433 cells compared to empty vector, as shown by the increase in cell viability. In contrast, CCL20 shRNA enhanced doxorubicin sensitivity of SKOV3 DR cells compared to control. CCL20 overexpression plasmid promoted NF-kB activation and positively regulated ABCB1 expression. Besides, ABCB1 overexpression plasmid enhanced the viability of SKOV3 and OVCA433 cells compared to empty vector under treatment with the same concentration of doxorubicin, whereas ABCB1 shRNA inhibited doxorubicin resistance of SKOV3 DR cells compared to control. In conclusion, CCL20 enhanced doxorubicin resistance of OC cells by regulating ABCB1 expression.

## Introduction

Ovarian cancer (OC), a tumor which develops in ovaries, is the most deathful gynecologic tumor with 10-year survival rate being ≤21% (Jelovac and Armstrong, 2011; Kalavska *et al.*, 2018). Although great efforts have been made to overcome OC, >150,000 patients are died of OC every year (Di Lorenzo *et al.*, 2018). There are many risk factors of OC, such as long period of ovulation (Kuznia and Roett, 2015), genetics (Norquist *et al.*, 2016), age (Gibson *et al.*, 2016), smoking (Jeon *et al.*, 2016) and so on. At early stage of OC, there is no specific symptom (Kuznia and Roett, 2015). However, OC patients at late stage may suffer from many discomforts, such as abdominal pain, pelvic pain, early satiety and so on (Kuznia and Roett, 2015). Chemotherapy is usually employed for general standard of OC care. Doxorubicin (as called adriamycin) belongs to anthracycline family and is one of chemotherapeutic agents often used for OC therapy. Doxorubicin can interfere with the function of topoisomerase II and then leads to cytotoxicity to tumor cells (Bodley *et al.*, 1989). Despite doxorubicin’s efficacy, doxorubicin resistance often occurs during the clinical treatment of OC and lead to chemotherapy failure. Hence, there is an urgent need to explore the mechanisms related to chemotherapy resistance of OC.

CCL20, also called LARC or MIP3A, is one of small cytokines and plays key roles in the development of various cancers, such as prostate cancer, breast cancer and so on (Beider *et al.*, 2009; Kim *et al.*, 2009). Moreover, Ignacio et al found that NF-κB-medicated CCL20 upregulation was involved in CXCR2-Driven OC development (Ignacio *et al.*, 2016). Son et al indicated that CCL20 may be one of the novel therapeutic targets for OC (Son *et al.*, 2013). However, the relationship between CCL20 expression and chemotherapy resistance of OC has yet to be studied.

Here, we established doxorubicin-resistant SKOV3 DR cells from SKOV3 cells via 6-month continuous treatment with gradient concentrations of doxorubicin. Quantitative PCR (qPCR) and Western blot analysis found that CCL20 mRNA and protein expression were enhanced in SKOV3 DR cells compared to that in SKOV3 cells. In the other hand, doxorubicin enhanced CCL20 expression in SKOV3 cells at time- and dose-dependent manner. Hence, we speculated that CCL20 expression may be positively linked with doxorubicin resistance of OC cells. The present study was undertaken to confirm the above hypothesis and the results showed that CCL20 facilitated doxorubicin resistance of SKOV3 and OVCA433 cells via modulating ABCB1 expression.

## Material and Method

### Cell lines and treatment

OC cell line SKOV3 and OVCA433 were bought from ATCC (Manassas, VA) and incubated in RPMI 1640 medium containing FBS and antibiotics. Doxorubicin-resistant SKOV3 DR cells were established from SKOV3 cells via 6-month continuous exposure to gradient concentrations of doxorubicin. In brief, SKOV3 cells were firstly treated with 0.5 μg/ml doxorubicin. After 3 weeks, doxorubicin concentration was increased to 1 μg/ml. Doxorubicin concentration was gradually increased up to 5 μg/ml.

### MTT assay

Cells (1,000 cells/well) were seeded in 24-well plates for 24 h, MTT solution (Sigma, St. Louis, MO) was added to each well. After 4 h, we aspirated supernatants and added DMSO to dissolve precipitated crystals. OD 490 value was detected.

### Cell count assay

1,000 cells were cultured in 6-well plates for 24 hours. Then, cells were dyed via trypan blue and macroscopically observed.

### Cell transfection

shRNAs targeting CCL20 or ABCB1 and negative control were bought from Invitrogen. Overexpressing plasmids of CCL20 or ABCB1 were constructed. Cells transfection was conducted via Lipofactamine 2000 (Invitrogen, Waltham, MA, USA).

### Western Blot

Proteins were extracted from cells, separated by electrophoresis and transferred to nitrocellulose membranes. After probing via specific antibodies against CCL20 (SantaCruz, SC-74234), NF-Κb (SantaCruz, SC-166588) and p-NF-κB (Abcam, Cambridge, MA, USA), ABCB1 (SantaCruz, SC-55510), membranes was analyzed via ECL detection system.

### QPCR and shRNA

Total RNAs were isolated from cells of different groups via TRIzol (Invitrogen, Waltham, MA, USA). Next, ~ 0.8 μg of each RNA sample was used to synthesize cDNA with Prime ScriptTM II 1st Strand cDNA Synthesis Kit (TaKaRa, Tokyo, Japan). QuantiFast^®^ SYBR^®^ Green PCR Kit (Qiagen, Hilden, Germany) was employed for qPCR and the PCR procedure was as follows: 1 cycle at 95°C for 5 min, 40 cycles at 95°C for 10 sec and at 60°C for 30 sec. Primers used for qPCR were listed below:

CCL20:

Forward 5'-CAGAAGCAGCAAGCAACT-3'

Reverse 5'-AGTCCAGTGAGGCACAAA-3';

ABCB1:

Forward 5'-cccatcattgcaatagcagg-3'

Reverse 5'-gttcaaacttctgctcctga-3'

GAPDH:

Forward 5'-ACC CAG AAG ACT GTG GAC TT-3'

Reverse 5'-TTC TAG ACG GCA GGT CAG GT-3'.

The shRNA sequence is below:

CCL20:

CCGGTGCTATCATCTTTCACACAAACTCGAGTTTGTGTGAAAGATGATAGCATTTTTG

ABCB1:

CCGGGCAGCAATTAGAACTGTGATTCTCGAGAATCACAGTTCTAATTGCTGCTTTTTG

### Statistical assay

Results were summarized as mean ± S.D. of at least 4 independent biological repeats. Data were analyzed via ANOVA or Student’s t-test and p<0.05 indicated significant difference.

## Results

### The establishment and identification of the doxorubicin resistant SKOV3 DR cell line

**Fig. 1:**
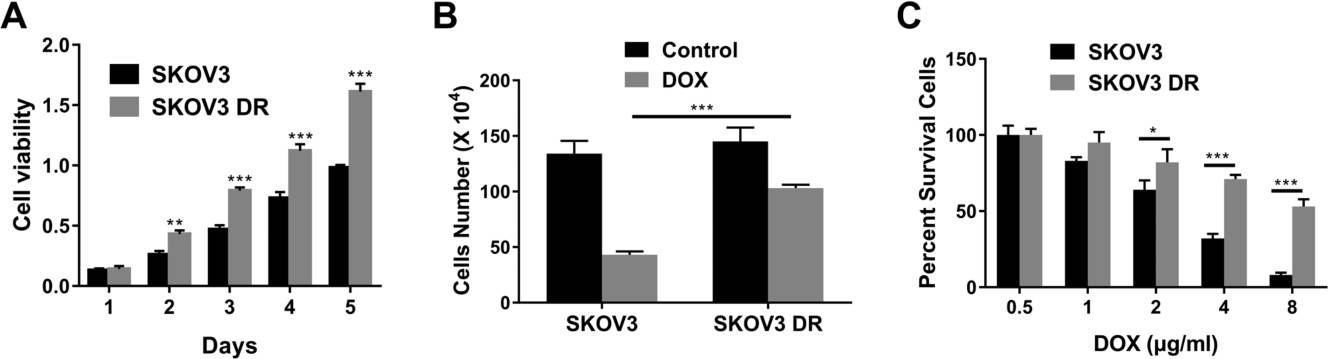
The establishment and identification of the doxorubicin resistant SKOV3 DR cell line. (A) MTT assay of SKOV3 DR or parental SKOV3 cells treated with 2 μg/ml doxorubicin at indicated time points. (B) Cell count assay of SKOV3 DR or parental SKOV3 cells treated with 2 μg/ml doxorubicin. (C) MTT assay of SKOV3 DR or parental SKOV3 cells treated with different concentrations of doxorubicin. Data are shown as mean±S.D. *P<0.05; **P<0.01; ***P<0.001; ns, not significant (Student’s t-test).

Fig. 1A showed that SKOV3 DR cells had higher viability under doxorubicin treatment compared to SKOV3 cells from the second day (p<0.01 for day 2 and p<0.001 for day 3–5). Fig. 1B showed that doxorubicin resistance of SKOV3 DR cells was enhanced compared to that of SKOV3 cells (p<0.001). Besides, Fig. 1C indicated that doxorubicin dose-dependently inhibited the viability of SKOV3 DR and SKOV3 cells, whereas SKOV3 DR cells were more resistant to doxorubicin (2, 4 and 8 μg/ml) than SKOV3 cells (p<0.001 for 4 and 8 μg/ml and p<0.05 for 2 μg/ml).

### CCL20 was upregulated in SKOV3 DR cells

**Fig. 2:**
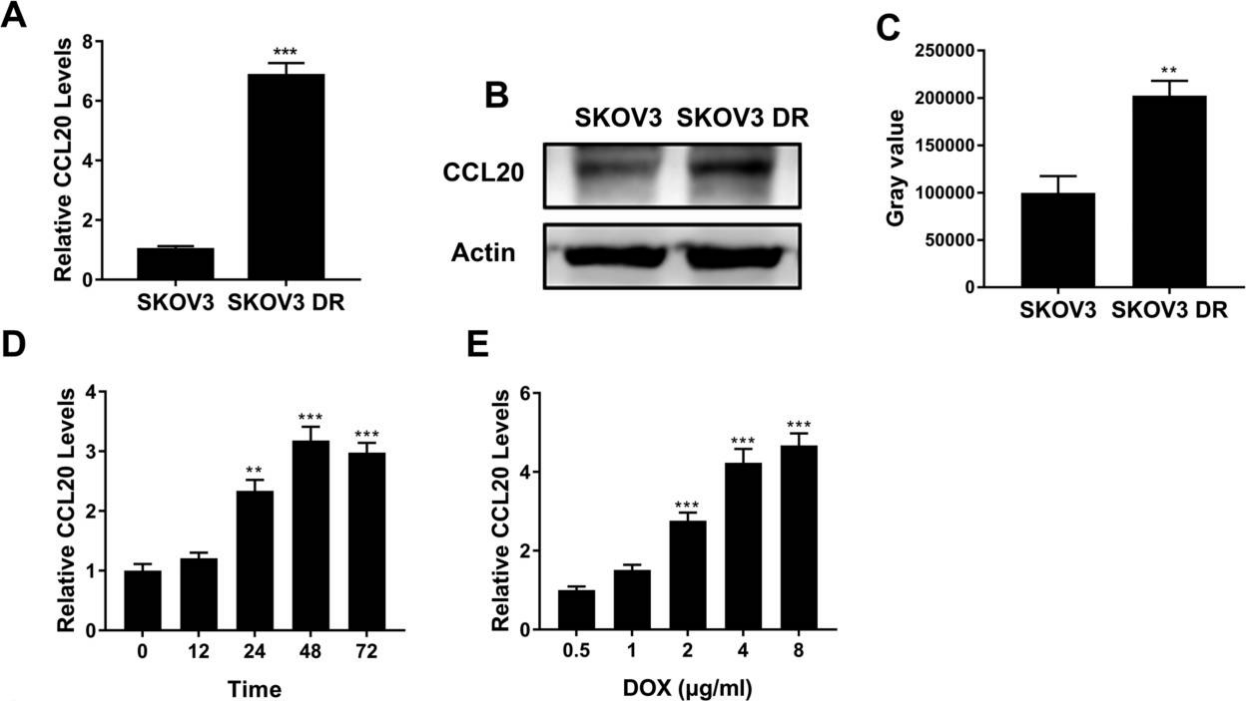
CCL20 was upregulated in SKOV3 DR cells. (A–B) QPCR (A) and Western blot (B) assay of CCL20 expression in SKOV3 and SKOV3 DR cells. (C) The grayscale analysis of the Western blot data in (B). (D) QPCR assay of CCL20 expression in SKOV3 cells treated with 2 μg/ml doxorubicin for different times. (E) QPCR assay of CCL20 expression in SKOV3 cells treated with different concentrations of doxorubicin for 48 hours. Data are shown as mean±S.D. *P<0.05; **P<0.01; ***P<0.001; ns, not significant (Student’s t-test in Fig. 2A and 2C, ANOVA test in Fig. 2D and 2E).

SKOV3 DR cells had higher mRNA (Fig. 2A, p<0.001) and protein level (Fig. 2B and C, p<0.01) of CCL20 than SKOV3 cells, respectively. Fig. 2D showed that doxorubicin time-dependently upregulated CCL20 expression in SKOV3 cells. At 24 h after treatment, CCL20 expression level in SKOV3 cells was increased to 257% of that in SKOV3 cells before treatment (Fig. 2D, p<0.01). In addition, Fig. 2E showed that doxorubicin dose-dependently upregulated CCL20 expression in SKOV3 cells. At 48 h after treatment, CCL20 expression level in SKOV3 cells treated with 2 μg/ml doxorubicin was increased to 280% of that in SKOV3 cells treated with 0.5 μg/ml doxorubicin (Fig. 2E, p<0.001).

### CCL20 was essential for doxorubicin resistance in OC

**Fig. 3:**
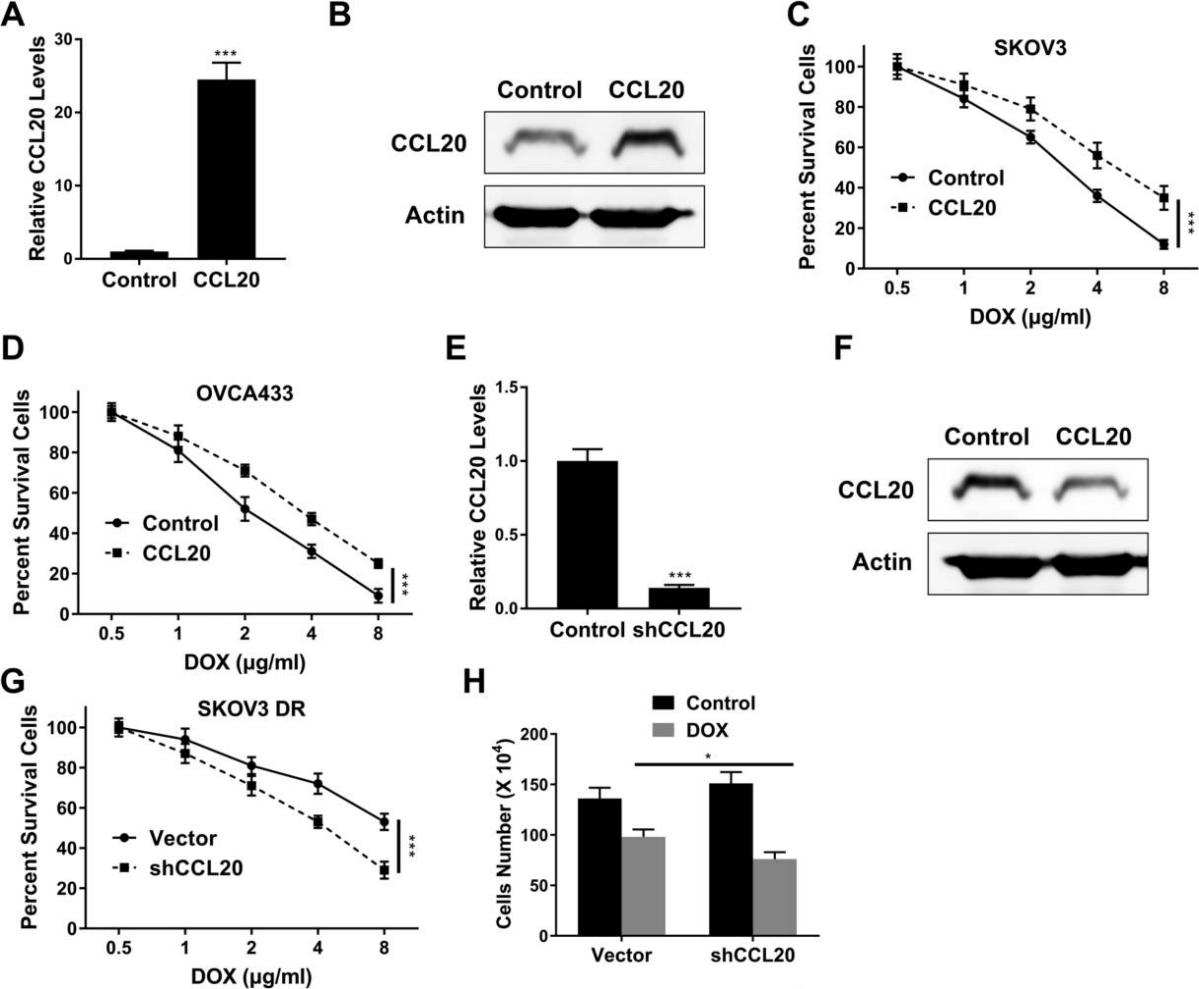
CCL20 was essential for doxorubicin resistance in OC cells. (A–B) QPCR (A) and Western blot (B) assay of CCL20 expression in SKOV3 cells transfected with CCL20 overexpression plasmid. (C–D) MTT assay of SKOV3 (C) and OVCA433 (D) cells transfected with CCL20 overexpression plasmid after doxorubicin treatment at indicated concentrations. (E–F) QPCR (E) and Western blot (F) assay of CCL20 expression in SKOV3 DR cells transfected with CCL20 shRNA. (G) MTT assay of SKOV3 DR cells transfected with CCL20 shRNA after doxorubicin treatment at indicated concentrations. (H) Cell count assay of SKOV3 DR cells transfected with CCL20 shRNA after 48-hour doxorubicin treatment (2 μg/ml). Data are shown as mean±S.D. *P<0.05; **P<0.01; ***P<0.001; ns, not significant (Student’s t-test in Fig. 3A, 3E and 3H, others ANOVA test).

Fig. 3A and 3B indicated that CCL20 overexpression plasmid significantly enhanced the mRNA (p<0.001) and protein expression of CCL20 in SKOV3 cells compared to control, respectively. MTT assay showed that the viability of SKOV3 (Fig. 3C) and OVCA433 (Fig. 3D) cells decreased as doxorubicin concentration increased, whereas CCL20 overexpression plasmid enhanced the viabilities of SKOV3 (Fig. 3C) and OVCA433 (Fig. 3D) cells compared to empty vector under treatment with the same concentration of doxorubicin (p<0.001).

In the other hand, Fig. 3E and 3F indicated that CCL20 shRNA significantly inhibited CCL20 mRNA (p<0.001) and protein expression in SKOV3 DR cells compared to control, respectively. Fig. 3G indicated that CCL20 shRNA inhibited the viabilities of SKOV3 DR cells compared to control under treatment with the same concentration of doxorubicin (p<0.001). In addition, cell count assay also showed that CCL20 shRNA enhanced doxorubicin sensitivity of SKOV3 DR cells compared to control (Fig. 3H, p<0.05).

### CCL20 activated NF-kB signal pathway to promote ABCB1 expression

**Fig. 4:**
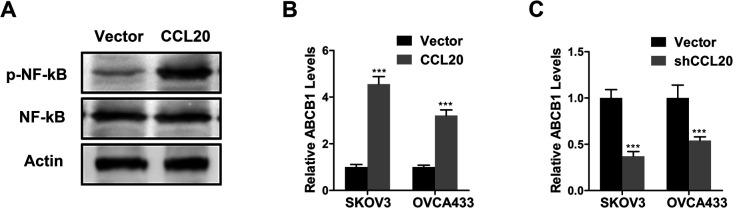
CCL20 actived NF-κB signal pathway to promote ABCB1 expression. (A) The activity of NF-κB signaling in SKOV3 cells transfected with CCL20 overexpression plasmid was determined by Western blot. (B–C) QPCR assay of ABCB1 expression in SKOV3 and OVCA433 cells transfected with CCL20 overexpression plasmid (B) or CCL20 shRNA (C). Data are shown as mean±S.D. *P<0.05; **P<0.01; ***P<0.001; ns, not significant (Student’s t-test).

Fig. 4A indicated that CCL20 overexpression plasmid promoted NF-kB phosphorylation compared to empty vector, whereas it had no effect on NF-kB expression in SKOV3 cells. QPCR assay showed that CCL20 overexpression plasmid enhanced ABCB1 expression in SKOV3 and OVCA433 cells (Fig. 4B, p<0.001). However, CCL20 shRNA inhibited ABCB1 expression in SKOV3 and OVCA433 cells (Fig. 4C, p<0.001).

### ABCB1 triggered drug resistance of OC cells

**Fig. 5:**
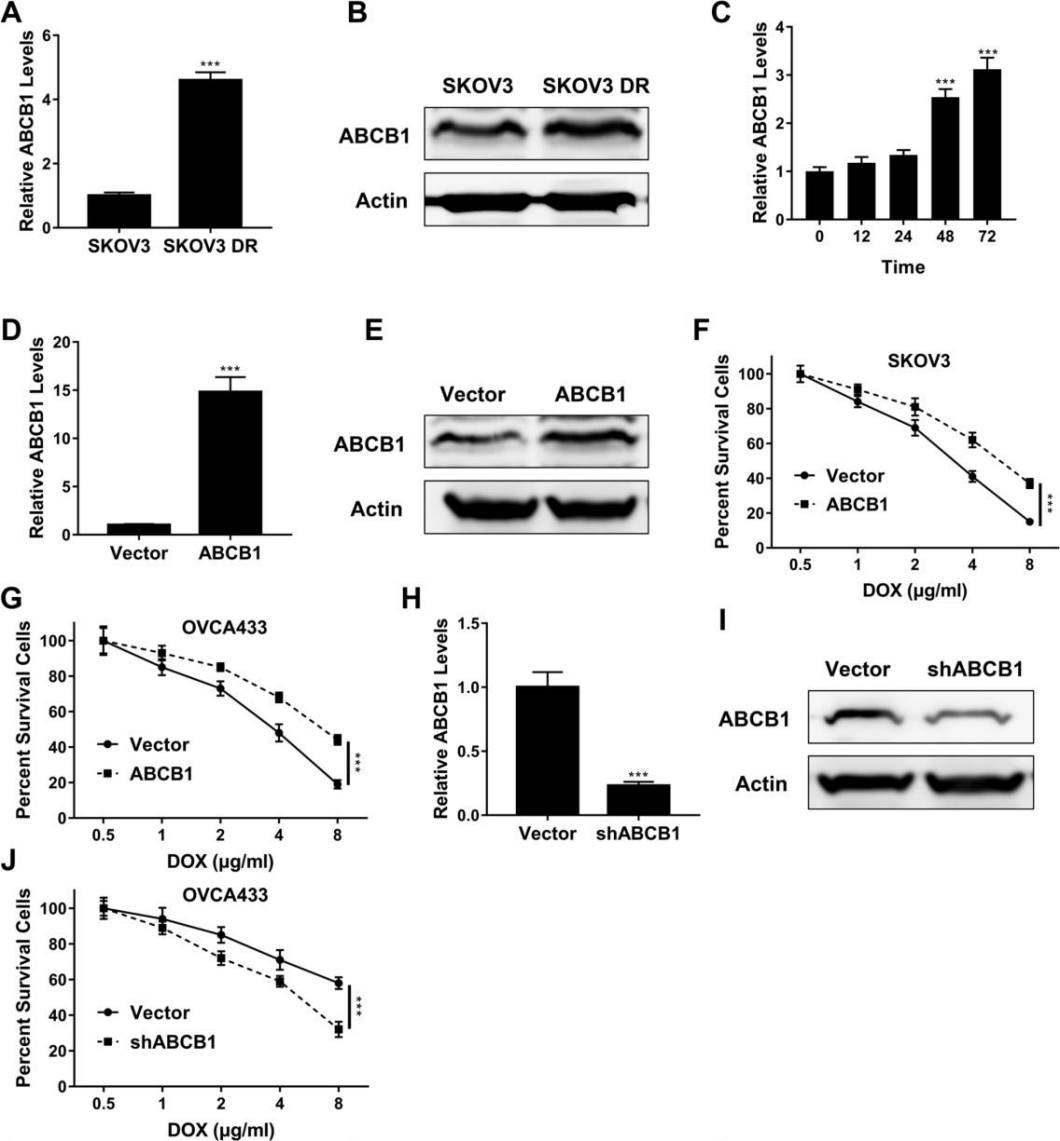
ABCB1 triggered drug resistance of OC cells. (A–B) QPCR (A) and Western blot (B) assay of ABCB1 expression in SKOV3 and SKOV3 DR cells. (C) QPCR assay of ABCB1 expression in SKOV3 cells treated with 2 μg/ml doxorubicin at indicated time points. (D–E) QPCR (D) and Western blot (E) assay of ABCB1 expression in SKOV3 cells transfected with ABCB1 overexpression plasmid. (F–G) Cell viability of SKOV3 (F) and OVCA433 (G) cells transfected with ABCB1 overexpression plasmid after doxorubicin treatment at indicated concentrations. (H–I) QPCR (H) and Western blot (I) assay of ABCB1 expression in SKOV3 DR cells transfected with ABCB1 shRNA. (J) MTT assay of SKOV3 DR cells transfected with ABCB1 shRNA after doxorubicin treatment at indicated concentrations. Data are shown as mean±S.D. *P<0.05; **P<0.01; ***P<0.001; ns, not significant (Student’s t-test in Fig. 5A, 5C and 5F, others ANOVA test).

Fig. 5A and 5B demonstrated that SKOV3 DR cells had higher mRNA (p<0.001) and protein level of ABCB1 than SKOV3 cells, respectively. Fig. 5C showed that doxorubicin time-dependently enhanced ABCB1 mRNA expression in SKOV3 cells. At 48 h, ABCB1 mRNA level in SKOV3 cells was increased 275% to that in cells before doxorubicin treatment (Fig. 5C, p<0.001). Next, OC cells were transfected with ABCB1 overexpression plasmid and we found that ABCB1 overexpression plasmid promoted ABCB1 mRNA (Fig. 5D, p<0.001) and protein (Fig. 5E) expression in SKOV3 cells compared to empty vector. MTT assay showed that ABCB1 overexpression plasmid enhanced the viability of SKOV3 (Fig. 5F) and OVCA433 (Fig. 5G) cells compared to empty vector under treatment via same concentration of doxorubicin (p<0.001). In the other hand, ABCB1 shRNA restricted ABCB1 mRNA (Fig. 5H, p<0.001) and protein (Fig. 5I) expression in SKOV3 DR cells compared to control. Moreover, ABCB1 shRNA inhibited the proliferation of SKOV3 DR cells compared to control under treatment via same concentration of doxorubicin (Fig. 5J, p<0.001).

## Discussion

Chemotherapy resistance is a severe phenomenon adopted by OC cells and brings major challenge to improve OC patients’ clinical outcomes, whereas the reasons for doxorubicin resistance of OC remain unknown. Here, SKOV3 cells were subjected to gradient concentrations of doxorubicin for 6 months. Subsequently, MTT and cell counts assay proved the successful establishment of doxorubicin-resistant SKOV3 DR cells. Further analysis showed that CCL20 mRNA and protein expression were upregulated in SKOV3 DR cells compared to SKOV3 cells. Moreover, doxorubicin time- and dose-dependently enhanced CCL20 expression in SKOV3 cells.

The aberrant expressions of chemokines were often found in various tumors (Gao and Fish, 2018; Karin, 2018; Rotondi *et al.*, 2018; Zhao *et al.*, 2018). CCL20 belongs to CC chemokine family and plays key roles in the development of different kinds of cancers. For example, Beider et al showed that the upregulation of CCL20 facilitated the development and aggressiveness of prostate cancer (Beider *et al.*, 2009). Kim *et al.* demonstrated that CCL20 expression level was positively linked with the aggressiveness of breast cancer cells (Kim *et al.*, 2009). Moreover, previous studies identified that CCL20 was upregulated in OC cells (Ignacio *et al.*, 2016; Son *et al.*, 2013). In addition, according to Chen *et al.*, CCL20 inhibition enhanced taxane sensitivity of breast cancer (Chen *et al.*, 2018). However, the relationship between CCL20 overexpression and the chemo-resistance of OC has not been explored. Here, our data proved that CCL20 overexpression plasmid inhibited doxorubicin sensitivity of OC cells, whereas CCL20 shRNA enhanced doxorubicin sensitivity of SKOV3 DR cells. Hence, the above data suggested that CCL20 may be essential for doxorubicin resistance of OC. In addition, chemokines may interact with their receptors and exhibit crucial roles in tumor development (Karin, 2018). Moreover, it is generally accepted that CCR6 is CCL20’s specific receptor (Wu *et al.*, 2007). Hence, further study should be done to find whether the dysregulation of CCR6 is involved in doxorubicin resistance of OC.

NF-κB, a transcription factor, plays key effects on cytokine and chemokines signaling (Son *et al.*, 2007) and is a crucial signaling for drug resistance of various tumors (Lin *et al.*, 2010). Moreover, recent studies showed that NF-κB activation was positively linked with drug resistance of various OC cells, such as A2780 (Zhao *et al.*, 2013) and SKOV3 (He *et al.*, 2017; Yan *et al.*, 2017) cells. In current study, we found that CCL20 overexpression plasmid promoted NF-κB activation in SKOV3 cells, which was consistence with previous studies (He *et al.*, 2017; Yan *et al.*, 2017). In addition, according to Zhao *et al.*, ABCB1-related chemo-resistance in paclitaxel-resistant OC cells can be inhibited through suppressing NF-κB activity (Zhao *et al.*, 2013). To find if the dysregulation of ABCB1 was associated with chemo-resistance of OC, SKOV3 and OVCA433 cells were transfected with CCL20 overexpression plasmid or CCL20 shRNA and qPCR assay showed that CCL20 overexpression plasmid promoted ABCB1 expression, whereas CCL20 shRNA inhibited ABCB1 expression in OC cells, suggesting that there was positive relationship between CCL20 and ABCB1 expression in OC cells.

It is generally accepted that the expression change of ABC membrane transporters is one of classical mechanisms of chemotherapy resistance in tumors (Chen *et al.*, 2016). ABCB1, also known as MDR1 or P-gp or CD243, belongs to ABC membrane transporter family and is an important multidrug resistance-related protein (Sharom, 2008). Previous studies prove that ABCB1 is a classical ATP-dependent drug efflux pump (Sharom, 2008) and plays key roles in the drug resistance of various tumors, such as gastric cancer (Wu *et al.*, 2018), urothelial cancer (Vallo *et al.*, 2017), lobular breast cancer (Krech *et al.*, 2012), osteosarcoma (Han and Shi, 2018) and so on. Moreover, increasing evidence shows that ABCB1 positively regulated OC’s resistance to different kinds of chemotherapy drugs, such as paclitaxel, olaparib, cisplatin, taxane and so on (Tian *et al.*, 2012, 2016; Vaidyanathan *et al.*, 2016). Therefore, we made a hypothesis that CCL20 may regulate doxorubicin resistance of OC cells through modulating ABCB1 expression.

To confirm the above speculation, ABCB1 mRNA level was evaluated and the results showed that ABCB1 was upregulated in SKOV3 DR cells compared to SKOV3 cells, and doxorubicin treatment time-dependently enhanced ABCB1 expression in SKOV3 cells. Moreover, further experiments confirmed that ABCB1 overexpression plasmid enhanced doxorubicin resistance of both SKOV3 and OVCA433 cells, as shown by the increase in cell viability. However, ABCB1 shRNA inhibited doxorubicin resistance of SKOV3 DR cells. Hence, ABCB1 triggered drug resistance of OC cells.

Taken together, we demonstrated that CCL20 facilitated OC chemotherapy resistance via modulating ABCB1 expression in vitro, as shown by the significant increase in cell viability under doxorubicin administration. Hence, the above findings suggested that both CCL20 and ABCB1 may be used as novel therapy targets to enhance clinical efficacy of doxorubicin treatment for OC patients. In addition, CCL20 and ABCB1 may be new potential markers for assessing doxorubicin resistance during OC clinical treatment.

CCL20 enhanced doxorubicin resistance of OC cells via regulating ABCB1 expression.

## Acknowledgments

None.

## Funding

None.

## Conflict of interest

None.

## References

[B1] Beider, K., Abraham, M., Begin, M., Wald, H., Weiss, I.D., Wald, O., et al. 2009. Interaction between CXCR4 and CCL20 pathways regulates tumor growth. PLoS One, 4: e5125. 19340288 10.1371/journal.pone.0005125 PMC2659745

[B2] Bodley, A., Liu, L.F., Israel, M., Seshadri, R., Koseki, Y., Giuliani, F.C., et al. 1989. DNA topoisomerase II-mediated interaction of doxorubicin and daunorubicin congeners with DNA. Cancer Res., 49: 5969–5978. 2551497

[B3] Chen, W., Qin, Y., Wang, D., Zhou, L., Liu, Y., Chen, S., et al. 2018. CCL20 triggered by chemotherapy hinders the therapeutic efficacy of breast cancer. PLoS Biol., 16: e2005869. 30052635 10.1371/journal.pbio.2005869 PMC6082578

[B4] Chen, Z., Shi, T., Zhang, L., Zhu, P., Deng, M., Huang, C., et al. 2016. Mammalian drug efflux transporters of the ATP binding cassette (ABC) family in multidrug resistance: A review of the past decade. Cancer Lett., 370: 153–164. 26499806 10.1016/j.canlet.2015.10.010

[B5] Di Lorenzo, G., Ricci, G., Severini, G.M., Romano, F., and Biffi, S. 2018. Imaging and therapy of ovarian cancer: clinical application of nanoparticles and future perspectives. Theranostics, 8: 4279–4294. 30214620 10.7150/thno.26345 PMC6134923

[B6] Gao, D. and Fish, E.N. 2018. Chemokines in breast cancer: Regulating metabolism. Cytokine, 109: 57–64. 29903574 10.1016/j.cyto.2018.02.010

[B7] Gibson, S.J., Fleming, G.F., Temkin, S.M., and Chase, D.M. 2016. The Application and Outcome of Standard of Care Treatment in Elderly Women with Ovarian Cancer: A Literature Review over the Last 10 Years. Front. Oncol., 6: 63. 27047797 10.3389/fonc.2016.00063 PMC4805611

[B8] Han, Z. and Shi, L. 2018. Long non-coding RNA LUCAT1 modulates methotrexate resistance in osteosarcoma via miR-200c/ABCB1 axis. Biochem. Biophys. Res. Commun., 495: 947–953. 29170124 10.1016/j.bbrc.2017.11.121

[B9] He, S., Niu, G., Shang, J., Deng, Y., Wan, Z., Zhang, C., et al. 2017. The oncogenic Golgi phosphoprotein 3 like overexpression is associated with cisplatin resistance in ovarian carcinoma and activating the NF-kappaB signaling pathway. J. Exp. Clin. Cancer Res., 36: 137. 28978336 10.1186/s13046-017-0607-0 PMC5628490

[B10] Ignacio, R.M., Kabir, S.M., Lee, E.S., Adunyah, S.E., and Son, D.S. 2016. NF-kappaB-Mediated CCL20 Reigns Dominantly in CXCR2-Driven Ovarian Cancer Progression. PLoS One, 11: e0164189. 27723802 10.1371/journal.pone.0164189 PMC5056735

[B11] Jelovac, D. and Armstrong, D.K. 2011. Recent progress in the diagnosis and treatment of ovarian cancer. CA Cancer J. Clin., 61: 183–203. 21521830 10.3322/caac.20113 PMC3576854

[B12] Jeon, S.Y., Go, R.E., Heo, J.R., Kim, C.W., Hwang, K.A., and Choi, K.C. 2016. Effects of cigarette smoke extracts on the progression and metastasis of human ovarian cancer cells via regulating epithelial-mesenchymal transition. Reprod. Toxicol., 65: 1–10. 27327412 10.1016/j.reprotox.2016.06.012

[B13] Kalavska, K., Minarik, T., Vlkova, B., Manasova, D., Kubickova, M., Jurik, A., et al. 2018. Prognostic value of various subtypes of extracellular DNA in ovarian cancer patients. J. Ovarian Res., 11: 85. 30243303 10.1186/s13048-018-0459-z PMC6196469

[B14] Karin, N. 2018. Chemokines and cancer: new immune checkpoints for cancer therapy. Curr. Opin. Immunol., 51: 140–145. 29579623 10.1016/j.coi.2018.03.004

[B15] Kim, K.Y., Baek, A., Park, Y.S., Park, M.Y., Kim, J.H., Lim, J.S., et al. 2009. Adipocyte culture medium stimulates invasiveness of MDA-MB-231 cell via CCL20 production. Oncol. Rep., 22: 1497–1504. 19885605 10.3892/or_00000593

[B16] Krech, T., Scheuerer, E., Geffers, R., Kreipe, H., Lehmann, U., and Christgen, M. 2012. ABCB1/MDR1 contributes to the anticancer drug-resistant phenotype of IPH-926 human lobular breast cancer cells. Cancer Lett., 315: 153–160. 22118813 10.1016/j.canlet.2011.09.038

[B17] Kuznia, A.L. and Roett, M.A. 2015. Genital Cancers in Women: Ovarian Cancer. FP Essent., 438: 24–30. 26569048

[B18] Lin, Y., Bai, L., Chen, W., and Xu, S. 2010. The NF-kappaB activation pathways, emerging molecular targets for cancer prevention and therapy. Expert Opin. Ther. Targets, 14: 45–55. 20001209 10.1517/14728220903431069 PMC3043547

[B19] Norquist, B.M., Harrell, M.I., Brady, M.F., Walsh, T., Lee, M.K., Gulsuner, S., et al. 2016. Inherited Mutations in Women With Ovarian Carcinoma. JAMA Oncol., 2: 482–490. 26720728 10.1001/jamaoncol.2015.5495 PMC4845939

[B20] Rotondi, M., Coperchini, F., Latrofa, F., and Chiovato, L. 2018. Role of Chemokines in Thyroid Cancer Microenvironment: Is CXCL8 the Main Player? Front. Endocrinol. (Lausanne), 9: 314. 29977225 10.3389/fendo.2018.00314 PMC6021500

[B21] Sharom, F.J. 2008. ABC multidrug transporters: structure, function and role in chemoresistance. Pharmacogenomics, 9: 105–127. 18154452 10.2217/14622416.9.1.105

[B22] Son, D.S., Parl, A.K., Rice, V.M., and Khabele, D. 2007. Keratinocyte chemoattractant (KC)/human growth-regulated oncogene (GRO) chemokines and pro-inflammatory chemokine networks in mouse and human ovarian epithelial cancer cells. Cancer Biol. Ther., 6: 1302–1312. 17712227 10.4161/cbt.6.8.4506

[B23] Son, D.S., Kabir, S.M., Dong, Y., Lee, E., and Adunyah, S.E. 2013. Characteristics of chemokine signatures elicited by EGF and TNF in ovarian cancer cells. J. Inflamm. (Lond.), 10: 25. 23800251 10.1186/1476-9255-10-25 PMC3694479

[B24] Tian, C., Ambrosone, C.B., Darcy, K.M., Krivak, T.C., Armstrong, D.K., Bookman, M.A., et al. 2012. Common variants in ABCB1, ABCC2 and ABCG2 genes and clinical outcomes among women with advanced stage ovarian cancer treated with platinum and taxane-based chemotherapy: a Gynecologic Oncology Group study. Gynecol. Oncol., 124: 575–581. 22112610 10.1016/j.ygyno.2011.11.022 PMC3278512

[B25] Tian, S., Zhang, M., Chen, X., Liu, Y., and Lou, G. 2016. MicroRNA-595 sensitizes ovarian cancer cells to cisplatin by targeting ABCB1. Oncotarget, 7: 87091–87099. 27893429 10.18632/oncotarget.13526 PMC5349973

[B26] Vaidyanathan, A., Sawers, L., Gannon, A.L., Chakravarty, P., Scott, A.L., Bray, S.E., et al. 2016. ABCB1 (MDR1) induction defines a common resistance mechanism in paclitaxel- and olaparib-resistant ovarian cancer cells. Br. J. Cancer, 115: 431–441. 27415012 10.1038/bjc.2016.203 PMC4985349

[B27] Vallo, S., Kopp, R., Michaelis, M., Rothweiler, F., Bartsch, G., Brandt, M.P., et al. 2017. Resistance to nanoparticle albumin-bound paclitaxel is mediated by ABCB1 in urothelial cancer cells. Oncol. Lett., 13: 4085–4092. 28599410 10.3892/ol.2017.5986 PMC5453046

[B28] Wu, X., Zheng, Y., Han, B., and Dong, X. 2018. Long noncoding RNA BLACAT1 modulates ABCB1 to promote oxaliplatin resistance of gastric cancer via sponging miR-361. Biomed. Pharmacother., 99: 832–838. 29710482 10.1016/j.biopha.2018.01.130

[B29] Wu, Y.-Y., Tsai, H.-F., Lin, W.-C., Hsu, P.-I., Shun, C.-T., Wu, M.-S., et al. 2007. Upregulation of CCL20 and Recruitment of CCR6(+) Gastric Infiltrating Lymphocytes in Helicobacter pylori Gastritis. Infection and Immunity, 75: 4357–4363. 17562763 10.1128/IAI.01660-06 PMC1951156

[B30] Yan, X.Y., Zhang, Y., Zhang, J.J., Zhang, L.C., Liu, Y.N., Wu, Y., et al. 2017. p62/SQSTM1 as an oncotarget mediates cisplatin resistance through activating RIP1-NF-kappaB pathway in human ovarian cancer cells. Cancer Sci., 108: 1405–1413. 28498503 10.1111/cas.13276 PMC5497928

[B31] Zhao, B.X., Sun, Y.B., Wang, S.Q., Duan, L., Huo, Q.L., Ren, F., et al. 2013. Grape seed procyanidin reversal of p-glycoprotein associated multi-drug resistance via down-regulation of NF-kappaB and MAPK/ERK mediated YB-1 activity in A2780/T cells. PLoS One, 8: e71071. 23967153 10.1371/journal.pone.0071071 PMC3744527

[B32] Zhao, H., Bo, Q., Wang, W., Wang, R., Li, Y., Chen, S., et al. 2018. CCL17-CCR4 axis promotes metastasis via ERK/MMP13 pathway in bladder cancer. J. Cell. Biochem., doi: 10.1002/jcb.27494. 10.1002/jcb.27494 30230587

